# Ultrasound Echo-Intensity Predicts Severe Pancreatic Affection in Cystic Fibrosis Patients

**DOI:** 10.1371/journal.pone.0121121

**Published:** 2015-03-24

**Authors:** Trond Engjom, Friedemann Erchinger, Birger N. Lærum, Erling Tjora, Odd H. Gilja, Georg Dimcevski

**Affiliations:** 1 Department of Clinical Medicine, University of Bergen, Bergen, Norway; 2 Department of Medicine, Haukeland University Hospital, Bergen, Norway; 3 Department of Medicine, Voss Hospital, Voss, Norway; 4 Department of Thoracic Medicine, Haukeland University Hospital, Bergen, Norway; 5 Department of Clinical Science, University of Bergen, Bergen, Norway; 6 Paediatric Department, Haukeland University Hospital, Bergen, Norway; 7 National Centre for Ultrasound in Gastroenterology, Haukeland University Hospital, Bergen, Norway; Klinikum rechts der Isar der TU München, GERMANY

## Abstract

**Background:**

Pancreatic destruction affects the majority of patients with cystic fibrosis. We aimed to relate ultrasound findings to exocrine pancreatic function and cystic fibrosis genotype.

**Methods:**

Patients with cystic fibrosis and a matched group of healthy controls were included. We performed transabdominal ultrasound, and recorded echo intensities of the pancreas and parenchymal characteristics according to endoscopic ultrasound based Rosemont criteria.

**Results:**

We included 39 patients and 29 healthy controls. The cystic fibrosis patients were grouped according to exocrine pancreatic function; Cystic fibrosis, insufficient (n = 20) and sufficient (n = 19). Echo intensity measures and visual score demonstrated hyper-echogenicity in the pancreas insufficient group compared to the pancreas sufficient groups (p<0.001). Ductal and parenchymal changes were not prevalent in any of the groups.

**Conclusion:**

The hyper-echoic pancreas was the most frequent ultrasonographic finding in exocrine pancreas insufficient cystic fibrosis patients. Pancreatic echo levels correlated to pancreatic phenotype.

## Introduction

Cystic fibrosis (CF) is an autosomal recessive disease caused by mutations in a single large gene on chromosome 7 encoding the cystic fibrosis transmembrane conductance regulator (CFTR) protein, a complex chloride channel and regulatory protein found in all exocrine tissues. The gene was discovered in 1989 and linked the disease to changes in the CFTR-protein [1–3]. The Cystic Fibrosis Mutation Database lists more than 1900 different mutations in the *CFTR* gene [4]. The prevalence in Scandinavian populations is reported to be 1 to 4–5000 live births [5]. Diagnostic criteria for cystic fibrosis are defined in the cystic fibrosis foundation consensus report [6].

Disturbed transport of chloride, sodium and bicarbonate leads to thick, viscous secretions in various organs, and increased salt content in sweat gland secretions. Patients with cystic fibrosis develop pancreatic damage as a result of defective ductal and acinar pancreatic secretion [7,8]. Population studies indicate that 72–88% of CF patients develop exocrine pancreatic insufficiency [9,10]. The main pathological findings in the pancreas of affected CF are atrophy, fibrosis and fatty infiltration [7,11]. Lately, the pancreatic insufficiency prevalence score has been suggested as a tool to predict pancreatic phenotype from the CF genotype [12,13].

CT and MRI studies of the pancreas in CF patients have demonstrated a signal intense pancreas with different patterns of fatty infiltration. (Diffuse fatty replacement, partial fatty replacement and pancreatic atrophy). Ductal changes, pancreatic cysts ranging from small to severe pancreatic cystosis, calcifications and hypoechoic areas representing fibrosis have also been demonstrated [14–18]. Transabdominal ultrasound is recommended regularly to detect cystic fibrosis liver disease [19]. Imaging of the pancreas with transabdominal ultrasound is a widespread and well-documented procedure [20]. Ultrasonography characteristics of the pancreas in cystic fibrosis pancreas are described in earlier studies, and the correlation to MRI findings is good [21–23]. Some earlier studies have demonstrated correlation between pancreatic function and radiological findings in patients with severe exocrine failure [18,23,24]. Neither of these studies performed assessment of exocrine pancreatic function by faecal elastase or direct pancreas function testing. In this study, we aim to correlate pancreatic ultrasound characteristics to CF genotype and exocrine pancreatic function assessed by secretin-stimulated endoscopic short test [25,26] or faecal elastase [10] in CF patients.

## Materials and Methods

### Subjects

**Fig 1:**
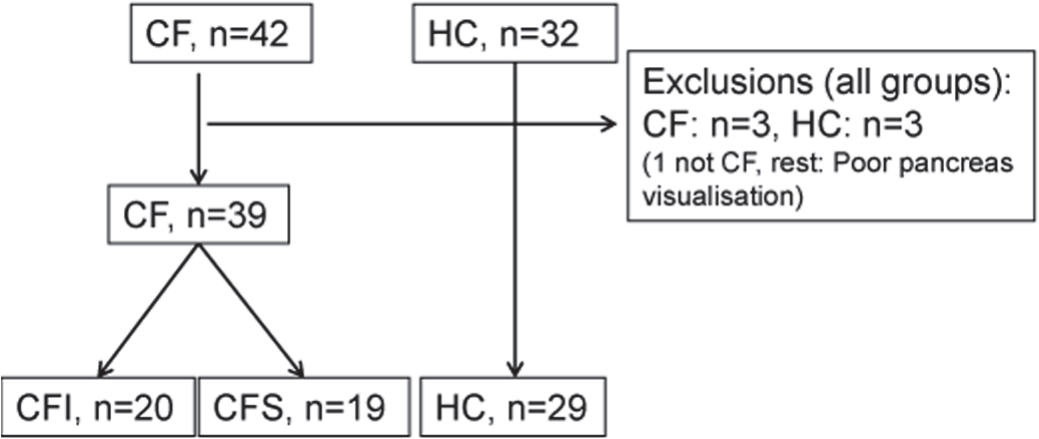
Inclusion flow chart. The figure displays the inclusion and exclusion of cystic fibrosis patients and healthy controls. (CF: Cystic fibrosis. CFI/CFS: Cystic fibrosis insufficient/ sufficient. HC: Healthy controls).

During a 4 year period (December 2010-May 2014), forty-two consecutive CF patients aged >15 years attending regular follow up in the CF clinic were offered a detailed evaluation of the pancreas. The CF diagnosis was evaluated according to the diagnostic criteria for cystic fibrosis [6]. An age and gender matched group of thirty-two healthy controls was also examined. The inclusion flow chart is displayed in Fig. 1. Lung-transplanted CF patients and subjects not able to give informed consent where not included in the study. Subjects with insufficient ultrasonographic visualization of the pancreas were excluded retrospectively. We excluded one CF patient due to lack of fulfilment of the diagnostic criteria. Further two were excluded due to poor pancreatic ultrasound visualization during repeated examinations. Three controls had poor ultrasonography visualization of the pancreas and were also excluded. We thus present results from 39 CF patients and 29 healthy controls (HC).

### Ethical considerations

The protocol was approved by the local ethics committee (Regional ethical committee western Norway. http://www.helseforskning.etikkom.no/. Mail: rek-vest@uib.no) Approval number: REK: 2010/2857-7) and the study was performed in accordance with the Helsinki Declaration. All subjects signed an informed consent to participate. The study included subjects from 15 years of age. For patients between 15 and 18 years consent was also signed by parents. The consent forms were approved by the local ethical committee. In the case of use of anonymized medical images, specific permission was obtained from the participants. Data underlying the conclusions in the study contain clinical information on humans and publication of the data material is subject to legal restrictions. A table containing anonymized raw data is published in the supplements of the article.

### Methods

#### Demographic data and genetics

Patient records were reviewed and patients and controls were interviewed. Age and sex of the patient, medication, smoking habits, alcohol consumption, body mass index, *CFTR* mutation status and sweat-test values (Na^+^ and Cl^-^) were documented. Genetic testing was performed using cystic fibrosis v3 Genotyping kit (Thirty-three *CFTR* mutations) or Elucigene CF-EU2 kit (Fifty-one mutations). Additional testing was done for the *CFTR* mutations 4005+2T>C and R117H. Cystic fibrosis patients with unknown mutation status after screening had whole gene sequencing for known *CFTR* mutations performed. Demographic data of the subjects are displayed in Table 1.

**Table 1:** Demographic data.

	Patients		Controls	P
	CFI (n = 20)	CFS (n = 19)	HC (n = 29)	
**Age** *	22(15–52)	22 (16–70)	29(18–66)	
**Gender** (♀/♂)	10 / 10	9 / 10	14 / 15	
**Body mass index**	21(20–23)	22(22–26)	22(21–26)	
**Sweat [Cl-]**	110(95–130)	72(67–79)	-	<0.001
**F-Elastase (μg/g)**	0.5 (0–14)	565(506–626)	-	<0.001
**D-bicarbonate (meq/L)**	13 (10–28)	119(109–127)	-	<0.001

Table listing the demographic data in the groups. Values in medians and IQ range. (CFI/CFS; Cystic fibrosis, pancreas insufficient/sufficient. HC; Healthy controls).

* Median (range).

Pancreatic insufficiency prevalence (PIP) corresponding to the least severe mutation was adapted from Ooi et Al [12,13]. Two mutations: 4005 + 2T<C: (3 of 7 PI), and S912x (3 of 3 PI) were not reported from Ooi, and thus calculated from our database. Pancreas insufficiency prevalence score in patients with only one or no known mutations were defined as zero.

#### Transabdominal ultrasound

The subjects were fasting >4 hours. A GE Logic E9 scanner with a 1–5 MHz curvilinear probe was used (GE Medical Systems and Primary Care Diagnostics, Milwaukee, WI, USA). We performed a complete ultrasound scanning with the subjects in supine position using a transverse or oblique epigastric probe position. The default abdomen configuration of the scanner was used to acquire the images: Frequency 4.0 MHz, dynamic range 66, frame rate 15–22 frames per second (varying). Pancreatic head area was traced covering the area from the pancreatic head to the level of the superior mesenteric artery in the pancreatic body. We evaluated ductal and parenchymal changes in the pancreas according to the endoscopic ultrasound (EUS) list of Rosemont criteria [27]. We also performed echo intensity (EI) measurements of the liver, pancreas and kidney and a closely related major vessel, using standard GE built-in software and the GE defined parameter Echo Level. Echo Level measures the mean intensity of pixels within a user defined area. The scanner utilizes the intensity data from raw data per pixel and calculates the average. Raw data pixel-measurements imply that there is no influence of gain, dynamic range or other scanner parameters. EL is measured in dB and is linear to the intensity [28]. The scale is defined through 255 gray levels reaching from white (Zero dB = Maximum intensity = Gray Level 255) to black (-99 dB = Minimum intensity = Gray Level 0)

**Fig 2:**
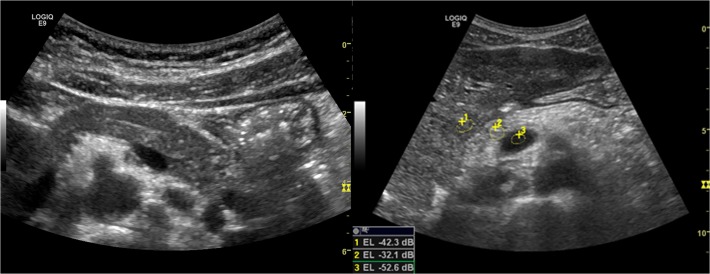
Pancreas ultrasonography. The figure is displaying a normal pancreas in pancreas sufficient patient (left) and a typical hyper echoic pancreas in an insufficient patient (Right). The Visual score graded echogenicity 1 for the left pancreas and 4 for the right pancreas. The image to the right display region of interest (ROI) for Echo intensity measures (1: Liver, 2: pancreas, 3: Vessel (superior mesenteric vein)).

Region of interest (ROI) was chosen at approximately the same tissue depths in the measures, avoiding vessels and hyper-echoic areas. Three measures were taken. The ratio between liver and pancreas echo intensity (LP SIR; liver pancreas signal intensity ratio) and vessel and pancreas (VP SIR; vessel pancreas signal intensity ratio) were calculated. (LP SIR = EI_liver_/ EI_pancreas_, VP SIR = EI_vessel_/ EI_pancreas_). (Fig. 2) The median ratio was chosen. A high ratio implicates a bright (Echo intensive) pancreas. We performed a visual score of echogenicity adapted from Worthen et Al [29]. Categories 1–4 are defined in Table 2. The score was set individually by three operators blinded to exocrine status and CF genotype. Median value was chosen. Discrepancies affecting absolute result were re-scored in consensus between the operators.

**Table 2:** Visual analogue scale (VAS).

Visual analogue scale	
**Grade 1**	Hypo-/ Iso-echogenic compared to liver
**Grade 2**	Slightly hyper-echogenic compared to liver
**Grade 3**	Marked hyper-echogenic compared to liver
**Grade 4**	Severe hyper-echogenic, equals retroperitoneal fat

Table defining the visual analogue scale for signal intensity after Worthen & Al (37).

#### Exocrine pancreas function

We assessed exocrine pancreatic function by a secretin-stimulated, short endoscopic function test described elsewhere [25]. Faecal elastase-1 was measured by a commercial analysis kit (ScheBo Biotech, Giessen, Germany). We classified patients as exocrine pancreatic sufficient or insufficient by secretin-stimulated bicarbonate concentration in duodenal juice. Patients with peak bicarbonate concentration <80meq/L were considered pancreas insufficient. Some cystic fibrosis patients had a dry tap or were unable to perform endoscopy. These were classified as insufficient by f-Elastase < 200μg/g.

### Statistical analysis

Statistics where calculated in SPSS statistics 22 (IBM SPSS Statistics, New York, USA) and SigmaPlot 11, (Copyright 2011 Systat Software Inc., San Jose, CA, USA). Normal distribution of the samples was tested by Kolmogorov-Smirnov test. We express the results as median values with range. Simple comparisons between groups were made by student t-tests or Mann-Whitney U-test as appropriate. Correlations were made by Spearman-Rank correlation tests. Accuracy data was calculated from receiver operator curves (ROC). Variance is expressed through 95% confidence intervals. 5% level of statistical significance was used. Interobserver reliability was calculated as intra-class correlation coefficients (ICC) in a random, two-way analysis. The scaled, ordinal data were analysed according to consistency. Categorical data were analysed according to complete agreement.

## Results

### Exocrine pancreatic function

When sorted by exocrine pancreatic function, patient groups were divided as follows: Cystic fibrosis, pancreatic insufficient (CFI): n = 20; cystic fibrosis pancreatic sufficient (CFS): n = 19. Data for faecal elastase and bicarbonate are displayed in Table 1.

### Pancreas ultrasound

#### Parenchymal and ductal characteristics

When we evaluated the pancreas according to criteria in the EUS based Rosemont chronic pancreatitis score, we found no major and only few minor criteria in our patients. One patient had severe cystic degeneration of the pancreas. Minor cysts of small diameters (<5mm), calcifications, severe ductal calibre variations were not seen. No minor or major criteria were detected in the healthy control group. There was good to excellent visualization of the head and body of pancreas in all the included patients.

#### Pancreas echo level

**Fig 3:**
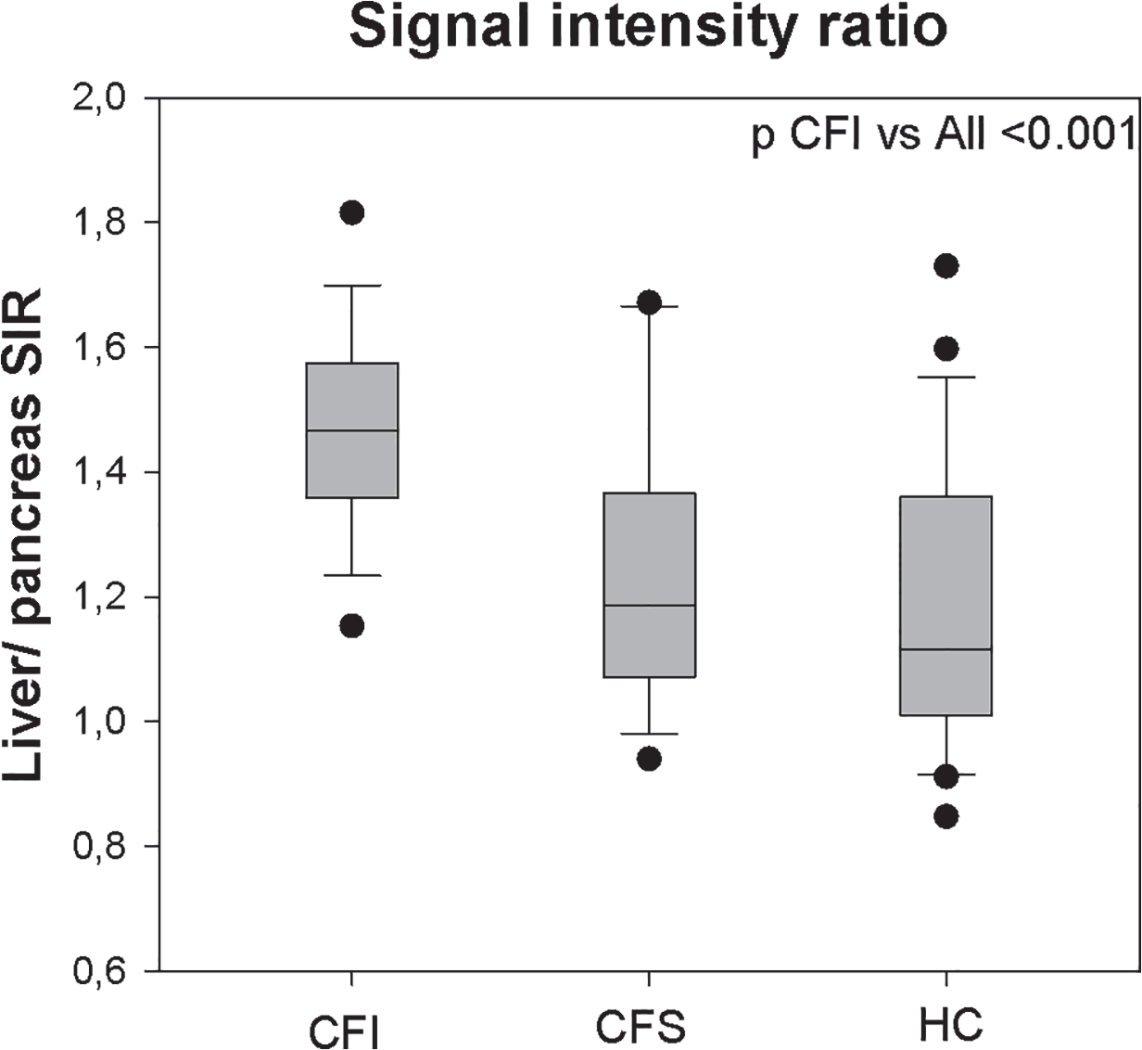
Liver-pancreas signal intensity ratio. Box plots displaying outliers, 95% confidence intervals, Interquartile ranges and median values for the liver pancreas signal-intensity ratio in corresponding groups. (SIR: Signal intensity ratio, CFI/CFS: Cystic fibrosis pancreas insufficient/ sufficient. HC: Healthy controls).

**Fig 4:**
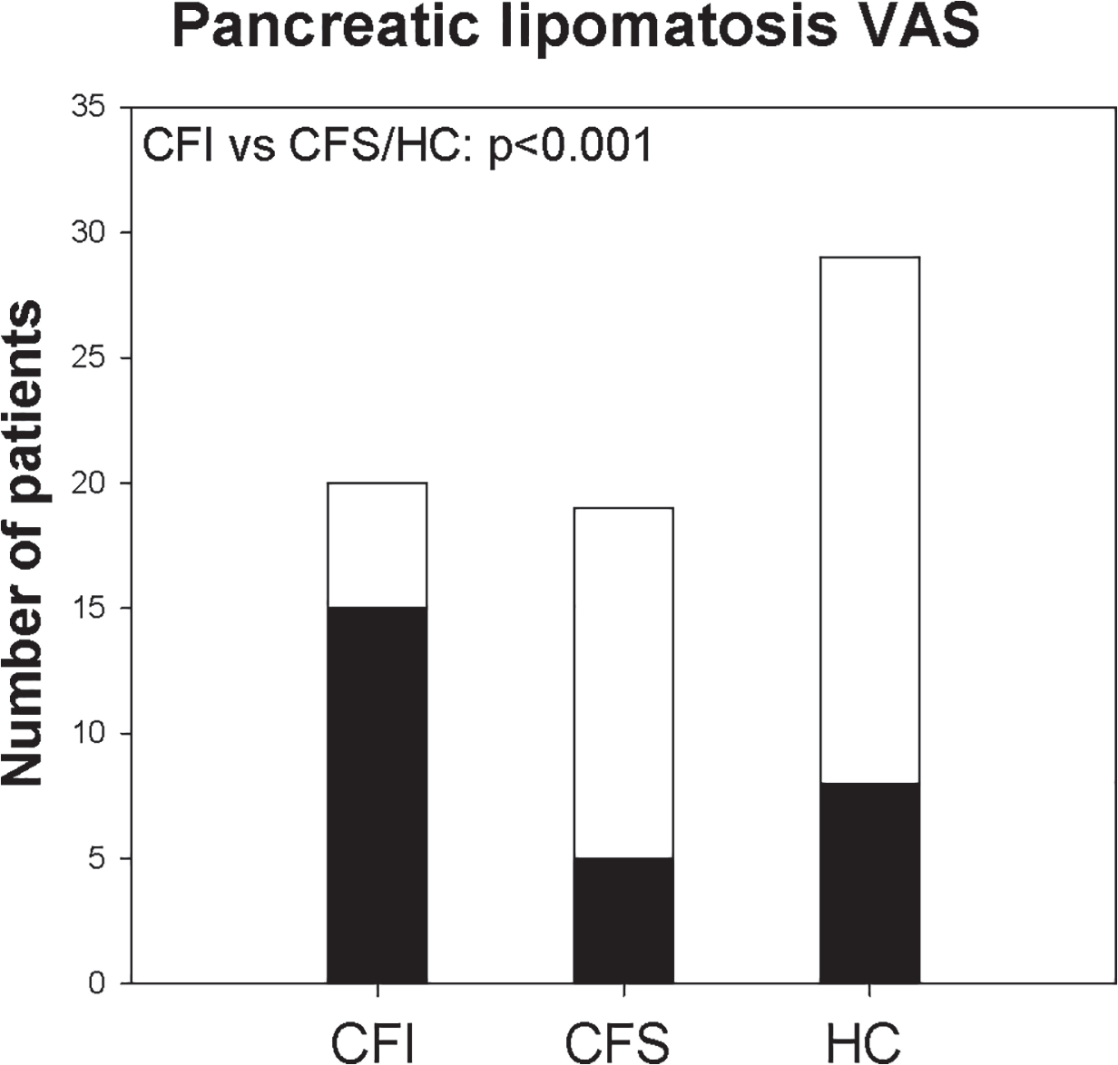
Lipomatosis VAS. Fig. displaying the rate of pancreas lipomatosis. White column display all patients in the groups. Dark columns display the number of subjects scored as VAS ≥3 (CFI/CFS: Cystic fibrosis pancreas insufficient/ sufficient. HC: Healthy controls).

The most frequent parenchymal characteristic was a homogenous, hyperechogenic pancreas (Fig. 2). Calculation of the closely related signal intensity ratios between liver and pancreas echo levels (LP SIR) and vessel and pancreas echo levels (VP SIR), both demonstrated significantly higher ratio in the CFI group compared to HC group and CFS group (p<0.001) (Fig. 3 and Table 3). The same differences were demonstrated by the Visual analogue score, where CFI group scored higher than CFS and HC group. Fig. 4 displays the proportion of VAS >2 in the different groups.

**Table 3:** Signal-intensity ratios.

	Patients		Controls	P
	CFI (n = 20)	CFS (n = 19)	HC (n = 29)	
**Liver-pancreas SIR**	1.47 (1.36–1.57)	1.19 (1.07–1.37)	1.12 (0.98–1.38)	<0.001
**Vessel-pancreas SIR**	1.76 (1.54–1.92)	1.39 (1.32–1.54)	1.41 (1.37–1.61)	<0.001

Table displaying echo intensity ratios. Values are expressed as medians (IQ range). (SIR; Signal intensity ratio).

Three of the patients in the CFI group and four of the patients in the HC group displaying hyperechoic pancreas were aged >45 years. We calculated Spearman rank correlations between age and Echo-level and VAS score. We did not find correlation between age and high echo levels in any of the groups.

Inter-operator agreement considering the VAS score was good with ICC for the average measures of 0.90 (0.84, 0.93), p<0.001. The agreement for the diagnosis of lipomatosis with a cut-off for VAS of <3 as normal, also was good. (ICC for average measures 0.85 (0.78, 0.90), p<0.001). Inter-agreement between the liver-pancreas echo intensity-measures were excellent with an ICC for the average measures of 0.98 (0.98, 0.99), p<0.001. Correlation (Spearman Ranks) between visual score and LP SIR was good. (r = 0.838, p<0.001).

We also performed ROC curves expressing the diagnostic quality of visual score and EL ratios predicting exocrine pancreatic failure. Sensitivity and specificity for the suggested cut-off for VAS and LP SIR are displayed in Table 4.

**Table 4:** Accuracy.

	Group	Sensitivity	Specificity	Cutoff	Accuracy
**VAS**	CF	0.79 (0.54–1.0)	0.88 (0.75–0.96)	VAS>2	0.83
**LP-SIR**	CF	0.89 (0.67–0.99)	0.74 (0.49–0.91)	1.25	0.81
**VP-SIR**	CF	0.81 (0.54–0.96)	0.81 (0.54–0.96)	1.54	0.90

Table displaying accuracy data for the ability of predicting pancreatic insufficiency using pancreas hyper echogenicity. Sensitivity and specificity data expressed as medians and 95% confidence intervals. VAS: Visual analogue scale, LP/VP-SIR: Liver/vessel-pancreas signal intensity ratio. Accuracy: Area under the curve calculated from receiver-operator curves).

#### Pancreatic size

Tracing of the area of the pancreatic head and body to the level of the of the superior mesenteric artery displayed smaller pancreas in both CF groups compared to Healthy controls (p<0.05).

### Genotype-phenotype considerations

There was a good correspondence between the predicted pancreas insufficiency prevalence and exocrine pancreatic function in our material. We also relate our findings of the hyperechoic pancreas to pancreas insufficiency prevalence. The results are displayed in S2 Table. When divided by our suggested LP SIR cut-off of 1.25, we found that the group displaying the hyperechoic pancreas has a markedly increased Pancreas insufficiency prevalence score compared to the non-hyperechogenic group (p<0.001).

## Discussion

In this study, we related features of transabdominal ultrasound of the pancreas to exocrine pancreatic function and genotype in CF patients. We demonstrated three main findings: First, we showed a higher pancreatic echogenicity, as a measure of pancreatic lipomatosis in pancreatic insufficient CF patients compared to pancreatic sufficient patients and healthy controls. Secondly, the same findings corresponded well to CF genotype and PIP score. Finally, we demonstrated that the presence of pancreatic lipomatosis in cystic fibrosis patients predicts exocrine pancreatic insufficiency in CF with an acceptable diagnostic accuracy. We did not demonstrate other ultrasonographic parenchymal characteristics to be prevalent.

The hyperechoic pancreas or pancreatic lipomatosis in cystic fibrosis estimated by both MRI and transabdominal ultrasound is described in earlier studies [15,17,18,22]. Relation of such findings to exocrine pancreatic failure assessed by precise and updated tests for exocrine pancreatic function is to our knowledge not yet demonstrated.

The study also includes novel considerations of diagnostic accuracy and intra- and inter-observer quality of ultrasound estimated lipomatosis. Pancreatic lipomatosis is a difficult finding to interpret due to the fact that it is not always related to pancreatic disease. Both age and obesity have been described factors associated with pancreatic lipomatosis in patients without pancreatic disease [29]. This explains the overlap of the phenomenon to the pancreas sufficient groups. We found more lipomatosis in the higher age group in our pancreas sufficient groups, but were not able to correlate the grade of lipomatosis to age in neither of the groups. Sub analysis (not presented) considering only CF patients below the age of 45 years and defining pancreatic insufficiency by the combination of both pathological F-elastase and D-bicarbonate reduces the overlap considerably. The fatty infiltration of the pancreas develops earlier in life in CF patients with affected pancreatic phenotype than in subjects without CF-induced pancreatic destruction. The hyperechogenic pancreas seems to predict pancreas affection in the age group from fifteen to forty five. The prevalence in CF patients younger than fifteen was not explored in this study.

Cystic fibrosis patients in western Norway were earlier described to have a lower grade of pancreatic failure than in other regions due to regional variations in genotype with higher prevalence of non-classical CF mutations [30]. This fact has given the opportunity to include an adequate number of pancreatic-sufficient cystic fibrosis patients in the study. A number of the included patients do not have a detected CF mutation. One patient was excluded after revision of the diagnosis due to weak CF phenotype; the others were included due to the presence of repeated positive sweat tests and significant clinical signs of CF.

We chose to use the endoscopic short test to define exocrine pancreatic function. By using this invasive direct hormonal stimulation test for exocrine insufficiency, we also aimed to detect patients with isolated decreased ductal function, not only end stage pancreatic disease. Using this definition, one more was classified as pancreas insufficient compared to the use of a combination of faecal elastase and duodenal bicarbonate. This patient was the only CFI patient below 45 without lipomatosis. A more conservative definition for pancreas insufficiency would strengthen the overall result.

Echo intensity ratio measures can be influenced by several factors. Setting of the region of interest, small depth incongruences between the related measures, shadowing from neighbouring organs and liver steatosis are the main factors to consider. Assessing vessel-pancreas ratio might be a way to exclude the variance in the liver standard due to liver-steatosis. We found that whether we chose a vessel or the liver as a comparing tissue, the EI-ratios, performed equally in the assessment of pancreatic lipomatosis. The main explanation of the little influence of the variations in the reference areas lies in the more pronounced variations in the pancreatic echo level. We conclude that the variations due to liver steatosis were of minor importance. The method performed excellent in repeated measures and we did not adjust for or exclude patients with liver steatosis. Still we recognize that the presence of fatty liver might reduce the value of the liver as a reference area in the liver-pancreas signal intensity ratio. Increased size of region of interest and better standardization in the placement of the ROI might improve performance of EI measurements.

Our VAS scale for visual evaluation has been used in earlier studies [29]. We found that there was a good correlation between EL ratio measures and the visual assessment, and that the inter-observer reliability was good. We found that estimate of lipomatosis by VAS scale performed largely equal to echo level measurements. Such an analogue scale has its limitations. We aimed to blind the VAS evaluation to knowledge of diagnosis and exocrine function, but still observer bias might influence the results.

Classical parenchymal and ductal findings have been demonstrated in MRI studies of the pancreas in cystic fibrosis patients. In one ultrasonographic study, the presence of small cysts was noted in 18% of the CF population [22]. Earlier autopsy studies have described the affected pancreatic tissue in CF consisting of micronoduli or cysts between 1 and 5 millimetres [7]. We were not able to demonstrate such findings. One explanation might be that if the pancreas is homogenously dominated by small lesions less than 1 millimetre, this will not present as single cysts, but rather a generally coarse parenchymal appearance. Cysts between 1 and 3 millimetres would be difficult to separate from pancreatic ducts. Cysts reaching 4–5 millimetres should have been detected. We conclude after careful review of the images combined with contrast enhanced ultrasound of the pancreas in the same patients (Unpublished data), that this is not a prevalent feature in our population of CF patients.

We are aware that the Rosemont criteria are validated for endoscopic ultrasound (EUS). EUS applies higher resolution and closer proximity to the pancreas, and often image more details than transabdominal ultrasound [27]. The ability to demonstrate minor criteria like stranding, minor main pancreatic duct calliper variations or dilated pancreatic duct side branches is limited using transabdominal ultrasonography. However, the visualization of the pancreas with transabdominal ultrasound in this patient group was excellent, and the detection of potential major criteria and minor criteria like cysts, hyperechoic foci and severe irregularities or dilatations of the main pancreatic ducts was demonstrated with fair sensitivity.

An explanation for the low grade of other pancreatic findings than pancreatic lipomatosis may be the lower prevalence of severe pancreatic genotypes in this study. Nevertheless, the group includes a sufficient number of classical ΔF508 CF patients with complete exocrine pancreatic failure to expect a higher prevalence of such findings if that was the case.

The Pancreas insufficiency prevalence score is a good tool to describe pancreatic genotype-phenotype relations. We believe that this score can prove to be of substantial aid dealing with CF patients where the genotype is known to detail. The demonstration of the excellent correspondence both to exocrine status and pancreatic ultrasonography findings in our study underlined the usefulness of the parameter both in science and practical use. The good correlation also underlines our message that pancreatic lipomatosis is a good predictor for pancreatic disease in CF patients.

## Conclusion

We conclude that the demonstration of the hyper echogenic pancreas predicts pancreatic affection in cystic fibrosis in the age group between fifteen and forty five years with good diagnostic accuracy. It may be that transabdominal ultrasound demonstrates inferiority to MRI and EUS regarding complete visualization of the organ, but the possibility of repeated examinations without radiation risk, the advantage of low costs and the lack of patient discomfort or harm still makes ultrasonography the most accessible imaging method for regular follow up of CF patients.

## Supporting Information

S1 Table Supporting information dataset. (XLSX)

S2 Table Supporting information dataset genotype. (DOCX)
