# Supplementary material for: Ultrasound Echo-Intensity Predicts Severe Pancreatic Affection in Cystic Fibrosis Patients
Source: PLoS One. 2015 Mar 24;10(3):e0121121. doi: 10.1371/journal.pone.0121121 (PMC4372286; doi:10.1371/journal.pone.0121121)
Supplement: S2 Table — (DOCX) [file pone.0121121.s002.docx]

**Table S2. Cystic fibrosis genotype.**

| **LP SIR>1.25 (n=23)** | (PIP) | **LP SIR<1.25 (n=16)** | (PIP) |
| --- | --- | --- | --- |
| F508del/ F508del (n=7) | (0.96) | F508del/ 4005 + 2T<C* | (0.43) |
| F508del/ G542X (n=2) | (0.96) | F508del/ 3849+10kbc>T | (0.09) |
| F508del/ S912x (n=3) | (0.96) | 621+1G>T/ 4005 + 2T<C | (0.43) |
| F508del/ E60x | (0.96) | G551D/ r75Q | (0) |
| F508del/ R117H | (0.04) | R117H/ Unknown | (0) |
| F508del/ 1525- 2A- >G | (0.96) | 1525- 47T- >G/ Unknown | (0) |
| F508del/ 4005 + 2T<C* | (0.43) | R117H/ Unknown | (0) |
| 394delTT/ S912x** | (1.00) |  |  |
| R1162x/ 1525- 2A- >G | (0.92) |  |  |
| F508del/ V232D | (0) |  |  |
| F508del/ Unknown | (0) |  |  |
| No mutation/ unknown (n=3) | (0) | No mutation/ unknown (n=9) | (0) |
| *PIP: 0.96 (0-1)* |  | *PIP: 0 (0-0.43)* |  |

Mutations detected split by the presence of hyper echoic pancreas (Split by liver-pancreas signal intensity ratio). Pancreas insufficiency prevalence score (PIP) given by the least severe mutation extracted from Ooi & Al [12,13] or calculated from our own population (LP SIR: Liver-pancreas signal intensity ratio. PI: Pancreas insufficient).

*4005 + 2T<C: 3 of 7 PI

** S912x: 3 of 3 PI.
